# Effect of Noise on Determining Ultrathin-Film Parameters from QCM-D Data with the Viscoelastic Model

**DOI:** 10.3390/s23031348

**Published:** 2023-01-25

**Authors:** Diethelm Johannsmann, Arne Langhoff, Christian Leppin, Ilya Reviakine, Anna M. C. Maan

**Affiliations:** 1 Institute of Physical Chemistry, Clausthal University of Technology, Arnold-Sommerfeld-Straße. 4, D-38678 Clausthal-Zellerfeld, Germany; 2 Advanced Wave Sensors S.L., Táctica Business Park, Algepsers Street, 24-1, 46988 Paterna Valencia, Spain; 3 Department of Bioengineering, University of Washington, Seattle, WA 98195-5061, USA; 4 Institute of Molecular Biology and Biotechnology (IMBB), 70013 Heraklion, Greece; 5 Polymer Science, Zernike Institute for Advanced Materials, University of Groningen, Nijenborgh 4, 9747 AG Groningen, The Netherlands

**Keywords:** thin soft films, film thickness, viscoelasticity, polymeric adsorbates, diffuse double layer, high-frequency rheology, quartz crystal microbalance with dissipation monitoring, QCM-D, PyQTM

## Abstract

Quartz crystal microbalance with dissipation monitoring (QCM-D) is a well-established technique for studying soft films. It can provide gravimetric as well as nongravimetric information about a film, such as its thickness and mechanical properties. The interpretation of sets of overtone-normalized frequency shifts, ∆*f*/*n*, and overtone-normalized shifts in half-bandwidth, ΔΓ/*n*, provided by QCM-D relies on a model that, in general, contains five independent parameters that are needed to describe film thickness and frequency-dependent viscoelastic properties. Here, we examine how noise inherent in experimental data affects the determination of these parameters. There are certain conditions where noise prevents the reliable determination of film thickness and the loss tangent. On the other hand, we show that there are conditions where it is possible to determine all five parameters. We relate these conditions to the mathematical properties of the model in terms of simple conceptual diagrams that can help users understand the model’s behavior. Finally, we present new open source software for QCM-D data analysis written in Python, PyQTM.

## 1. Introduction

The quartz crystal microbalance (QCM) is a convenient tool for film thickness determination [1]. Following Sauerbrey [2], films applied to a resonator surface in air or a vacuum decrease the resonance frequency in proportion to their mass per unit area. For sufficiently stiff films, the Sauerbrey relationship also applies in liquids [3]; however, the layer thickness is not always the parameter of prime importance to an experimentalist. Advanced QCMs, often termed “QCM-D” for “quartz crystal microbalance with dissipation monitoring”, report a shift in half-bandwidth, ΔΓ, in addition to a shift in frequency, Δ*f*, and they do so for a number of different overtones. Overtones are usually labeled by their overtone order, *n* (often *n* = 3, 5, 7, 9, 11). The half-bandwidth, Γ, is related to the “dissipation factor”, *D*, as Γ = *Df*_res_/2 The additional information contained in the sets of Δ*f*/*n* and ΔΓ/*n* gives access to certain nongravimetric parameters [4,5], such as the mechanical properties of the film. 

**Figure 1:**
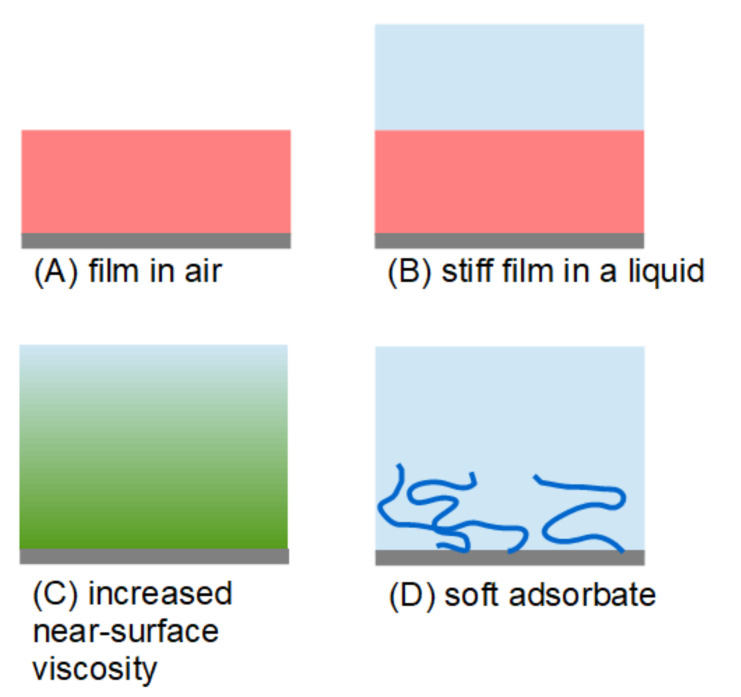
A set of configurations in which the interpretation proceeds in different ways. For cases (**A**,**B**) the values of Δ*f*/*n* and ΔΓ/*n* can be interpreted, respectively, at least approximately. For systems with increased near-surface viscosity (**C**), Δ*f*/*n* and ΔΓ/*n* have an interpretation, but the width of the range, inside which the viscosity is increased, is no longer accessible. Interpretation is difficult for soft adsorbates (**D**).

There is an established formalism with which to predict Δ*f* and ΔΓ from the thicknesses and viscoelastic parameters of planar films [6,7,8,9]. The algebra is readily extended to multilayers or even samples with continuous viscoelastic profiles (functions *G*′(*z*) and *G*″(*z*), with *G*′ and *G*″ being the real and imaginary parts of the shear modulus, respectively [5]). Modeling in the forward direction is easy, but the inversion can be nontrivial. The following text sticks to, firstly, planar layers and, secondly, to the mathematics. The text identifies cases in which the derivation of the viscoelastic parameters is possible. It describes the recipes and states the conditions under which these rules apply. We are only concerned with single layers with a thickness much below the wavelength of shear sound, λ. The wavelength can be many microns for dry polymer films. For liquid-like samples, λ is largely equivalent to the penetration depth of the shear wave, δ, which is about 200 nm for 5 MHz resonators in water. “Thin soft layers” in this context are thinner than about 5 nm (depending on the softness). Figure 1 provides an overview of the configurations that will be discussed in the following sections. These are as follows:

(A) A thin film in air;
(B) A stiff, thin film in a liquid;
(C) A semi-infinite Newtonian liquid with slightly altered viscosity close to the resonator surface;
(D) A soft film in a liquid.

Case C differs from cases A, B, and D in Figure 1, in that the sample is characterized by a smooth viscoelastic profile. It does not constitute a film. The profile has a characteristic length which takes the role of the thickness, but is not strictly the same as a film thickness.

**Figure 2:**
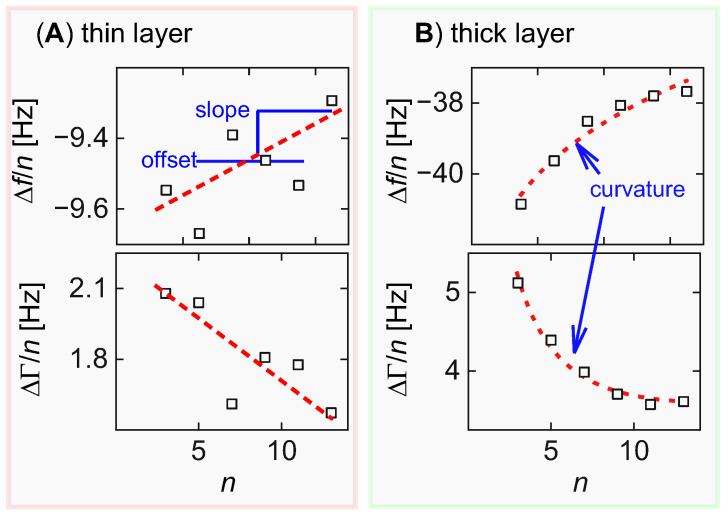
For very thin films (**A**) the fractional noise is too large to let a curvature in plots of Δ*f*/*n* and ΔΓ/*n* versus *n* be determined reliably. Interpretation must rely on the offsets and the slopes for Δ*f*/*n* and ΔΓ/*n* (totaling four parameters). If the model contains five parameters the problem is underdetermined. For thicker films (**B**) the curvatures can be determined reliably; thus, five model parameters can also be derived reliably.

Case A is well-behaved. For thin films in air there is a well-known procedure to derive the softness of a film from QCM data [10,11]. For cases B and C viscoelastic parameters can only be derived if assumptions are made. In case B the assumption amounts to a stiff film [12]. In case C the assumption is that the layer is almost a Newtonian liquid. Of course, these assumptions must be justified. The soft adsorbate (case D) is problematic. In short, the problem is underdetermined. As discussed in Section 2, five parameters are needed in order to describe a viscoelastic layer. This is where the noise in QCM-D experiments on thin films creates a problem: the curvatures in plots of Δ*f*/*n* and ΔΓ/*n* vs. *n* need to be determined reliably (Figure 2). Where this is impossible, the interpretation fails. In the example shown in Figure 2 (the adsorption of a diblock co-polymer), the reliable determination of the curvatures is only possible if the layer thickness is larger than ~10 nm. This value depends on the experimental details, and on the experimental noise in particular.

“Noise” in this context includes irregular differences between different experiments and crystals, which may be larger than the statistical noise in one single experiment. Presumably, these go back to poorly controlled effects of compressional waves, the latter being caused by small admixtures of flexural motion to the thickness shear deformation [13,14]. These vary between crystals, but not over time during one experiment. The magnitude of such irregularities is in the range of 0.1 Hz (see Figure 2A). One can fight these irregularities to some extent by measuring the viscosity of the liquid prior to the adsorption experiment. One compares the frequency shifts in the wet state to those in the dry state and fits the difference with the Kanazawa–Gordon equation [15]. A bad crystal can be recognized at this time (and be discarded) based on outliers in this simple experiment.

Again, extensive averaging will not reduce the absolute values of these irregularities because they are constant in time; however, the fractional errors become smaller if the overall signal increases. In Figure 2B the magnitude of the irregularities is similar to those in Figure 2A, but they now are superimposed onto a QCM response of 40 Hz, as opposed to 10 Hz in Figure 2A. To be more precise, the n-dependence of Δ*f*/*n* spans 3 Hz in Figure 2B, while it spans 0.3 Hz in Figure 2A. The viscoelastic properties of the layer are inferred from the *n*-dependence of Δ*f*/*n* and ΔΓ/*n*. With films thicker than about 10 nm, the fractional errors are small enough to derive six robust parameters from a set of Δ*f*/*n* and ΔΓ/*n*, which suffices to fix the five free parameters of the model. Otherwise, only four such robust parameters (offsets and slopes in plots from Δ*f*/*n* and ΔΓ/*n* vs. n) can be derived, which do not allow the inference of five model parameters.

Two caveats to the above are as follows:

- Data from the fundamental often show irregular and erratic behavior. The fundamental is therefore usually discarded.
- The QCM produces artifacts on the high overtones when applied to samples which are known to be Newtonian liquids [16]. The imaginary part of the viscosity as reported by the QCM is sometimes negative, which is unphysical.

## 2. Target Parameters of a Viscoelastic Analysis

Different parameters are in use to quantify viscoelastic responses. The shear modulus, $\tilde{G}$ = *G*^′^ + i*G*^″^, is the ratio of shear stress to shear strain. In the following, the tilde denotes a complex parameter (mostly a viscoelastic response function). $\tilde{G}$ is popular in polymer research [17]. The shear compliance, $\tilde{J}$ = 1/$\tilde{G}$ = *J*′− i*J*″, is the ratio of strain to stress. QCM-D experiments are most easily analyzed in terms of *J*’ and *J*″ because the trivial case (Sauerbrey-type behavior) corresponds to $\tilde{J}$ = 0 (rather than *G*′ = ∞ or G″ = ∞). Additionally, the recipes from Section 3.2 and Section 3.3 relate *J*′ and *J*″ to the characteristic features of the plots as opposed to *G*′ and *G*″. The viscosity, $\tilde{\eta }$ = $\tilde{G}$/(iω) = η′ − iη″, is useful when the layer under study has a viscosity of η′ ≈ η′_bulk_ and small elasticity (η″ ≈ 0). The bulk in the following is assumed to be a Newtonian liquid (η′ = const, η″ = 0). In all three cases ($\tilde{G}$, $\tilde{J}$, or $\tilde{\eta }$), the real part and the imaginary part may be replaced by absolute values (|$\tilde{G}$|, |$\tilde{J}$|, or |$\tilde{\eta }$|) and the loss tangent (tan δ = *G*″/*G*′ = *J*″/*J*′ = η′/η″). The loss tangent is actually independent of whether the quantification of viscoelasticity occurs with $\tilde{G}$, $\tilde{J}$, or $\tilde{\eta }$. If the loss tangent has a peak at some frequency, the medium under study undergoes relaxations with rates similar to the frequency of the peak in tan δ.

**Figure 3:**
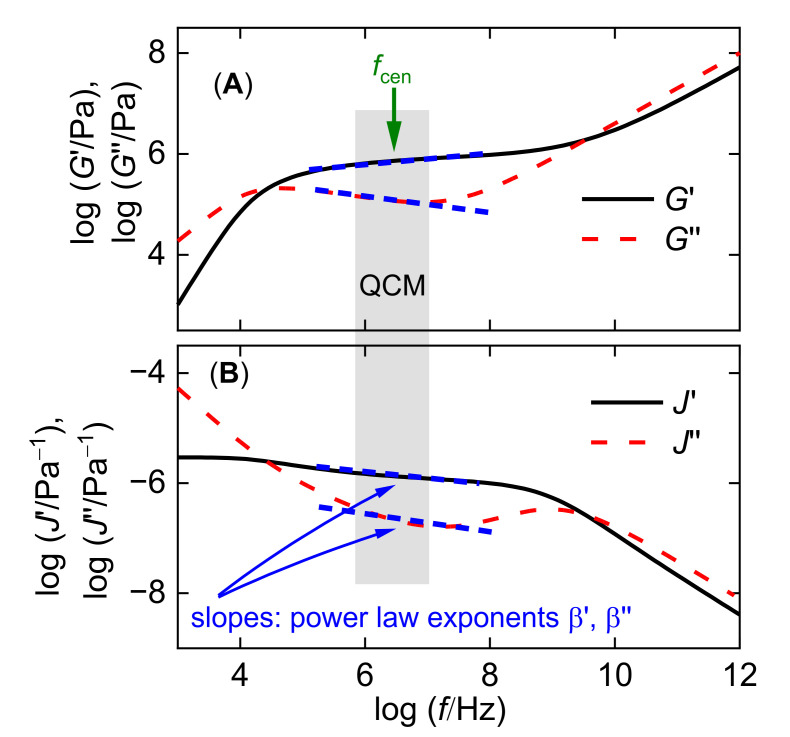
The shear moduli of viscoelastic materials depend on frequency. The plot shows a typical rheological spectrum of a solution of a long-chain linear polymer. The frequency scale extends over many decades, while the QCM only covers about one decade. In this limited frequency range, *G*′(*f*) and *G*″(*f*) can be approximated by power laws (dashed blue lines) in panel A. For the QCM, the representation with {*J*′(*f*), *J*″(*f*)} in panel B is more practical than the set {*G*′(*f*), *G*″(*f*)}. The graph discusses viscoelasticity in terms of either {*G*ʹ, *G*ʹʹ} or {*J*′, *J*″}. The same arguments apply to—for instance—the pair {|$\tilde{G}$|, tan δ} (see Equation (1)).

In soft matter, the viscoelastic parameters depend on frequency (Figure 3). This might lead to the impression that $\;{2n}_{\mathrm{ovt}}+1$ parameters were needed to predict the set {Δ*f*/*n*, ΔΓ/*n*}. These would be *G*′ and *G*″ at each of the *n*_ovt_ frequencies, plus the layer thickness. The problem of inversion would then be underdetermined because the experiment only reports 2*n*_ovt_ parameters; however, viscoelastic spectra are usually smooth. In the limited frequency range covered by the QCM, |$\tilde{G}$| and tan δ (*f*) can be approximated with fair accuracy by power laws (Figure 3). These are of the following form:

(1) $$\left|G\left(f\;\right)\right|\;\approx \;\left|G_{\mathrm{cen}}\right|{\left(\frac{f}{f_{\mathrm{cen}}}\right)}^{\beta^{\prime }}\;\tan\;\delta \left(f\right)\;\approx \;{\left(\tan\delta \right)}_{\mathrm{cen}}{\left(\frac{f}{f_{\mathrm{cen}}}\right)}^{\beta^{\prime \prime }}$$

*f*_cen_ is a frequency in the center of the QCM’s range (often *f*_cen_ ≈ 30 MHz). |*G*_cen_| and (tan δ)_cen_ are the values at this frequency. If *J*′ and *J*″ are used rather than |$\tilde{G}$| and tan δ, one may write the following:

(2) $$J^{\prime }\left(f\right)\;\approx {J^{\prime }}_{\mathrm{cen}}{\left(\frac{f}{f_{\mathrm{cen}}}\right)}^{\beta^{\prime }}\;J^{\prime \prime }\left(f\right)\;\approx \;{J^{\prime \prime }}_{\mathrm{cen}}{\left(\frac{f}{f_{\mathrm{cen}}}\right)}^{\beta^{\prime \prime }}$$

Of course, the values of the power law exponents, $\beta^{\prime }$ and $\beta^{\prime \prime }$, differ between Equations (1) and (2). Ideally, the inversion of QCM data with respect to the viscoelasticity of a sample produces a set of five parameters, which are the thickness, two parameters describing a material’s stiffness at *f* = *f*_cen_, and two power law exponents. The stiffness at *f*_cen_ may be quantified with the pair {|$\tilde{G}$|, tan δ}, quantified with the pair {*J*′, *J*″}, or with some other pair. Given that viscoelastic dispersion cannot be ignored for soft matter (β ≠ 0, β ≠ 0), any realistic model taking viscoelasticity into account is bound to have at least five free parameters. Fewer parameters amount to assumptions.

## 3. Background

### 3.1. Underlying Equations

For a single film, the standard model for viscoelastic planar films on a QCM surface predicts the following [6,7,8,9]:

(3) $$\frac{\Delta f+i\Delta \Gamma }{f_{0}}=\frac{-{\tilde{Z}}_{f}}{\pi Z_{q}}\cdot \frac{{\tilde{Z}}_{f}\tan({\tilde{k}}_{f}d_{f})-i{\tilde{Z}}_{bulk}}{{\tilde{Z}}_{f}+i{\tilde{Z}}_{bulk}\tan({\tilde{k}}_{f}d_{f})}-\frac{i{\tilde{Z}}_{bulk}}{\pi Z_{q}}$$

$\tilde{k}$ = ω/$\tilde{c}$ = ω/($\tilde{G}$/ρ)^1/2^ is the wave number; $\tilde{Z}$= (ρ$\tilde{G}$)^1/2^ is the shear wave impedance; the subscripts “f” and “q” denote the film and the resonator, respectively; and *f*_0_ is the frequency of the fundamental. Equation (3) applies to all thicknesses, at least in principle. It does not involve a Taylor expansion in film thickness, *d*_f_. (It does, however, make use of the small-load approximation [5].) Equation (3) was used to produce the fits shown in Section 3.5.

### 3.2. Determination of Softness Is Possible for Thin Films in Air

High-frequency rheology on thin films in air is well-established [5,10,11]. If the ambient medium is air (if $\tilde{Z}$_bulk_ *=* 0), Equation (3) simplifies to the following:

(4) $$\frac{\Delta f+i\Delta \Gamma }{f_{0}}=\frac{-{\tilde{Z}}_{f}}{\pi Z_{q}}\tan({\tilde{k}}_{f}d_{f})$$

Taylor expanding Equation (4) to the third order in *d*_f_ results in the following:

(5) $$\frac{\Delta f+i\Delta \Gamma }{n}\;\approx \;-\frac{m_{f}}{m_{q}}f_{0}\left[1+\frac{{\left(n\pi \right)}^{2}}{3}\left(\frac{{J_{f}}^{\prime }-i{J_{f}}^{\prime \prime }}{\rho_{f}}Z_{q}^{2}\right){\left(\frac{m_{f}}{m_{q}}\right)}^{2}\right]$$

*m*_f_ (the mass per unit area) is equal to ρ_f_ *d*_f_. *m*_q_ = *Z*_q_/(2*f*_0_) is the mass per unit area of the resonator plate. For the reasons discussed in Ref. [18], a slightly better approximation is as follows:

(6) $$\frac{\Delta f+i\Delta \Gamma }{n}\;\approx \;-\frac{m_{f}}{m_{q}}f_{0}\left[1+\frac{{\left(n\pi \right)}^{2}}{3}\left(\frac{{J_{f}}^{\prime }-\;i{J_{f}}^{\prime \prime }}{\rho_{f}}Z_{q}^{2}\;-\;1\right){\left(\frac{m_{f}}{m_{q}}\right)}^{2}\right]$$

The additional term of −1 is not of importance in the following. The separation of the real and imaginary parts leads to the following:

(7) $$\frac{\Delta f\;}{n}=-\frac{m_{f}}{m_{q}}f_{0}\left[1+\frac{{\left(n\pi \right)}^{2}}{3}\left(\frac{{J_{f}}^{\prime }}{\rho_{f}}Z_{q}^{2}\;-\;1\right){\left(\frac{m_{f}}{m_{q}}\right)}^{2}\right]\;\frac{\Delta \Gamma }{n}=-\frac{m_{f}}{m_{q}}f_{0}\frac{{\left(n\pi \right)}^{2}}{3}\left(\frac{{J_{f}}^{\prime \prime }}{\rho_{f}}Z_{q}^{2}\;-\;1\right){\left(\frac{m_{f}}{m_{q}}\right)}^{2}$$

**Figure 4:**
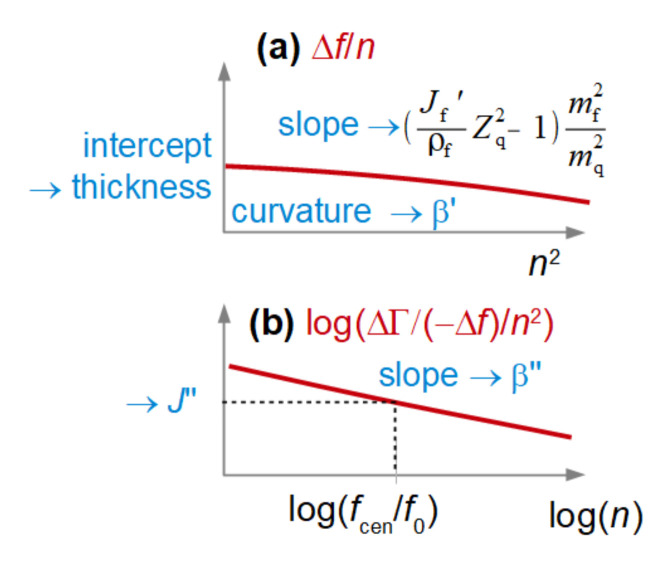
For a thin film in air, the set of parameters {*d*_f_, *J*_f_′, *J*_f_″, β′, β″} can be derived from plots of Δ*f*/*n* versus *n*^2^ (**a**) and log(ΔΓ/(−Δ*f*)/*n*^2^) versus log(*n*) (**b**), as shown above. Arrows indicate which system parameters are derived from certain features of the plot.

For a thin film in air (case A in Figure 1), there is a recipe on how to proceed with deriving the entire set of parameters {*d*_f_, *J*_f_′, *J*_f_″, β′, β″} from an experiment. The error bar on βʹ may be substantial (see the third bullet point below), but the error occurs on only this one parameter. Because the errors are not cross-correlated, the values of the other parameters remain robust. The scheme is sketched in Figure 4. The rules are based on Equation (5).

The recipe is as follows:

- The film thickness, *d*_f_ (more precisely, the mass per unit area, *m*_f_), is obtained from a plot of Δ*f*/*n* versus *n*^2^ (Figure 4A). A line (possibly with a slight curvature) is fitted to the data. The intercept of this line with the y-axis is proportional to *m*_f_. More specifically, one has *m*_f_ = −Δ*f*_intercept_/(*nf*_0_)*m*_q_.
- The elastic compliance, *J*_f_′, is obtained from the slope of this line.
- The power law exponent of the elastic compliance, β′, is obtained from the curvature of this line. This curvature is often determined with considerable uncertainty. The error bars on β’ are correspondingly large.
- The viscous compliance, *J*_f_″, is obtained from the ratio between the bandwidth shift and the frequency shift following the relationship below:

(8) $$\frac{\Delta \Gamma }{-\Delta f}\;\approx \;\frac{1}{3}\left({J_{f}}^{\prime \prime }\frac{Z_{q}^{2}}{\rho_{f}}\right){\left(\frac{{n\pi m}_{f}}{m_{q}}\right)}^{2}$$

Equation (8) follows from Equation (5), where the real part of the right-hand side was approximated as unity. ΔΓ/(−Δ*f*) in this case is not independent of the thickness (more precisely, of the mass per unit area, *m*_f_). It rather scales as *m*_f_^2^. This is not a problem, because *m*_f_ is determined with fair accuracy from the intercept with the y-axis in a plot of Δ*f*/*n* vs. *n*^2^; it may be a problem when *m*_f_ is small. Nongravimetric effects are difficult to detect for layers which are only a few nanometers thick. These layers shear under their own inertia, and the inertial forces are weak for thin films. On the positive side, the approximation underlying Equation (8) (namely that the real part of the right-hand side in Equation (6) is about unity) can always be reached by making the film thin enough.

- The power law exponent of the viscous compliance, β″, is obtained from the slope in a log–log plot of ΔΓ/(−Δ*f*)/*n*^2^ versus *n* (Figure 4B).

Following ref. [19] (but slightly deviating from it), we call ΔΓ/(−Δ*f*) the “acoustic ratio”. In ref. [19] the acoustic ratio is defined as Δ*D*/(−Δ*f*/*n*), with Δ*D* being the shift in the dissipation factor. For 5 MHz crystals, the two parameters differ by a factor of 2.5 (meaning that ΔΓ/*n* = (2.5 Hz/ppm) Δ*D*). If defined as ΔΓ/(−Δ*f*), the acoustic ratio is independent of the fundamental frequency of the resonator and is also dimensionless.

The relationships presented in the above recipe and illustrated in Figure 4 are not exact; they are presented here for the purpose of illustration. Accurate values of the various parameters are obtained from a fit with Equation (3). The recipes are more transparent than the fitting procedure and offer users a conceptual view of how the fitting procedure works.

### 3.3. Determination of Elastic Softness Is Possible for Thin, Stiff Films in a Liquid

For a film in a liquid (cases B–D in Figure 1), the analysis is more complicated because of the influence of the bulk. Taylor expanding Equation (3) to the first order in *d*_f_ (with $\tilde{Z}$_bulk_ ≠ 0) results in the following:

(9) $$\frac{\Delta f+i\Delta \Gamma }{n}=-\frac{2f_{0}^{2}}{Z_{q}}\rho_{f}d_{f}\left[1-\frac{{\tilde{Z}}_{bulk}^{2}}{{\tilde{Z}}_{f}^{2}}\right]$$

For a film in a liquid, there is a recipe similar to Section 3.2., but this recipe only applies if the film is sufficiently stiff. One starts from Equation (9), replaces ${\tilde{Z}}_{f}^{-2}$ by ${\tilde{J}}_{f}$/ρ_f_, and replaces ${\tilde{Z}}_{bulk}^{2}$ by iωρη_bulk_:

(10) $$\begin{array}{cc} \frac{\Delta f+i\Delta \Gamma }{n} & \approx -\frac{2f_{0}^{2}}{Z_{q}}\rho_{f}d_{f}\left[1-\frac{{\tilde{J}}_{f}}{\rho_{f}}i\omega \eta_{bulk}\right]\\ & \approx \;-\frac{{2f}_{0}^{2}}{Z_{q}}{\rho d}_{f}\left[1\;-\;\left({J_{f}}^{\prime }-i{J_{f}}^{\prime \prime }\right){i\omega \eta }_{\mathrm{bulk}}\right] \end{array}$$

In the second step, the density was assumed as constant (ρ_f_ ≈ ρ_bulk_ ≈ ρ). Because Equation (10) is linear in thickness, it also holds in an integral sense:

(11) $$\frac{\Delta f+i\Delta \Gamma }{n}\approx -\frac{2f_{0}^{2}}{Z_{q}}\rho \int_{0}^{\infty }\left[1-{\tilde{J}}_{f}(z)i\omega \eta_{bulk}\right]dz$$

The integral in Equation (11) amounts to a characteristic length, which takes the role of the film thickness should the sample not be a film with a sharp interface to the bulk. Whether or not the viscoelastic profile is well-approximated by a box profile ($\tilde{J}$(*z*) = const = $\tilde{J}$_*f*_ inside the film) is sometimes questionable. The QCM will always report apparent parameters because it does not recognize the profile.

If $\tilde{J}$_*f*_ in Equation (10) is zero, it reproduces the Sauerbrey result. If the layer is noticeably soft, the viscoelastic correction (the term in square brackets) lowers the value of −Δ*f*/*n*. This decrease in apparent thickness (in thickness naively derived with the Sauerbrey equation) is sometimes called the “missing-mass effect” [20].

In the limit of a thin, stiff film, the acoustic ratio is as follows:

(12) $$\frac{\Delta \Gamma }{-\Delta f}\;\approx {\;\omega \eta }_{\mathrm{bulk}}{J^{\prime }}_{f}{=2\pi nf}_{0}\eta_{\mathrm{bulk}}{J^{\prime }}_{f}$$

In this limit, the acoustic ratio is independent of film thickness, and depends only on the viscoelastic properties of the layer. Equation (12) follows from Equation (10), separated into its real and imaginary parts:

(13) $$\frac{\Delta f}{n}=\frac{-{2f}_{0}^{\;2}}{Z_{q}}m_{f}\left[1\;-\;n\left({2\pi f}_{0}\frac{\rho_{\mathrm{bulk}}}{\rho_{f}}\eta_{\mathrm{bulk}}\right){J_{f}}^{\prime \prime }\right]$$

(14) $$\frac{\Delta \Gamma }{n}=\frac{{2f}_{0}^{\;2}}{Z_{q}}m_{f}\left({n\;2\pi f}_{0}\frac{\rho_{\mathrm{bulk}}}{\rho_{f}}\eta_{\mathrm{bulk}}\right){J_{f}}^{\prime }$$

Dividing Equation (14) by Equation (13) leads to the following:

(15) $$\frac{\Delta \Gamma }{-\Delta f}\;\approx \;\frac{n\left({2\pi f}_{0}\frac{\rho_{\mathrm{bulk}}}{\rho_{f}}\eta_{\mathrm{bulk}}\right){J_{f}}^{\prime }}{1\;-\;n\left({2\pi f}_{0}\frac{\rho_{\mathrm{bulk}}}{\rho_{f}}\eta_{\mathrm{bulk}}\right){J_{f}}^{\prime \prime }}\;\approx \;\frac{{\omega \eta }_{\mathrm{bulk}}{J_{f}}^{\prime }}{1\;-{\;\omega \eta }_{\mathrm{bulk}}{J_{f}}^{\prime \prime }}$$

If the denominator can be replaced by unity, Equation (12) is recovered. The condition allowing for this simplification can be phrased as *J*_f_″<< *J*_bulk_″ or, equivalently, as η_f_′>>η_bulk_. Again, Equation (12) only applies if this condition is met.

**Figure 5:**
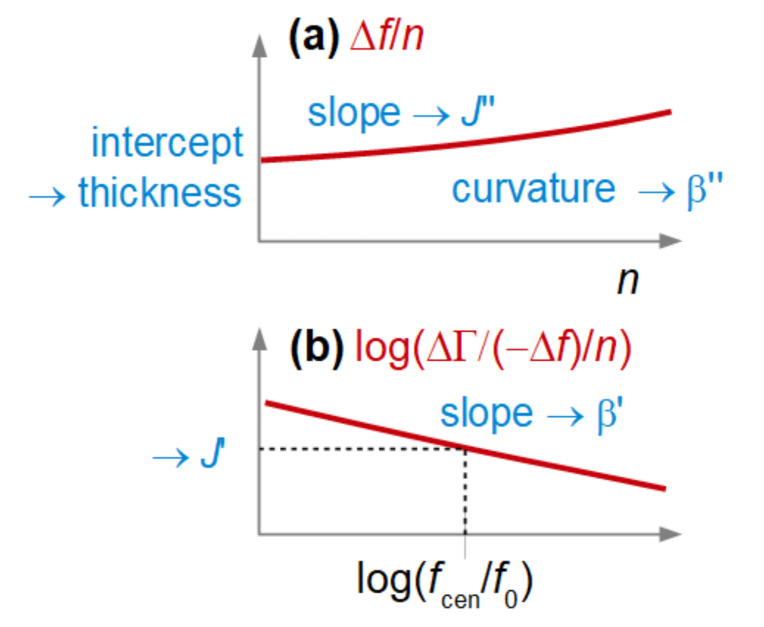
A thin, stiff film in a liquid also allows viscoelastic parameters from the sets of Δ*f*/*n* and ΔΓ/*n* to be extracted. The plot of Δ*f*/*n* versus *n* yielded the thickness and the viscous compliance (**a**). The plot of log(ΔΓ/(−Δ*f*)/*n*) versus log(*n*) yields the elastic compliance and the power-law exponent β′ (**b**). Arrows indicate which system parameters are derived from certain features of the plot.

If η′_f_ >> η_bulk_, the recipe to derive viscoelastic parameters is as follows (see Figure 5):

- The film thickness, *d*_f_, is obtained from a plot of Δ*f*/*n* versus *n*. A line (possibly with a slight curvature) is fitted to the data. The intercept of this line with the y-axis is proportional to the thickness.
- The viscous compliance, *J*_f_″, is obtained from the slope of this line. Note: For the dry film, the elastic compliance is derived from the slope. It is the viscous compliance here.
- The power law exponent of the viscous compliance, β″, is obtained from the curvature of this line. Error bars are often large because the curvature is determined with poor accuracy.
- The elastic compliance, *J*_f_′, is obtained from the acoustic ratio, following Equation (12).
- The power law exponent of the elastic compliance, β′, is obtained from the slope in a log–log plot of ΔΓ/(−Δ*f*)/*n* versus *n* (Figure 5B).

Again, the recipes and their visualization in Figure 5 are presented to illustrate how the fitting procedure works, while accurate values of the film parameters are obtained through fitting frequency and bandwidth shifts to Equation (3) with an appropriate value of $\tilde{Z}$_bulk_.

We [21] and others [19,22], have, in the past, offered empirical interpretations of the acoustic ratio for planar as well as structured films. For example, Tsortos et al. studied surface-attached DNA and concluded that the acoustic ratio was related to the intrinsic viscosity of the layer [19]. Srimasorn et al. have studied a series of glucosaminoglucans in much detail and concluded that—for this homologous series of molecules—the acoustic ratio was proportional to the molecular weight [22]. There were deviations from proportionality at the high end, but the relationship was still monotonous. Tellechea et al., for adsorbates of a particulate nature, plotted the acoustic ratio versus −∆*f*/*n*, where −∆*f*/*n* was a substitute for coverage. These plots revealed straight lines, which for all overtones extrapolated to the same point on the x-axis. Moreover, the value of −∆*f*/*n* at the intercept corresponded to the diameter of the adsorbed particles (after conversion to a thickness with the Sauerbrey equation). The procedure is intriguing insofar as a parameter, which usually counts as gravimetric (the layer thickness), was extracted from a set of parameters, which usually count as nongravimetric (the acoustic ratios) [21]. For the moment, it remains unclear how the geometric parameters, as empirically inferred from the acoustic ratio, relate to the viscoelastic model discussed here. Particulate adsorbates are outside of the scope of the current work. We are only concerned with homogeneous planar layers.

### 3.4. Increased Near-Surface Viscosity (Case C in Figure 1)

The fact that Sauerbrey-type behavior in liquids may originate from processes not at all related to adsorption is noticed in experiments with an electrochemical QCM (EQCM) [23]. An EQCM often targets electrodeposition, caused by charge transfer across the electrode surface [24]; however, the diffuse double layer also makes a contribution to the frequency shift [25]. The problem is not severe as long as the layer formed by electrodeposition is thicker than about 10 nm. It is a problem, though, in studies on underpotential deposition [26,27], where the layer thickness corresponds to about a monolayer.

**Figure 6:**
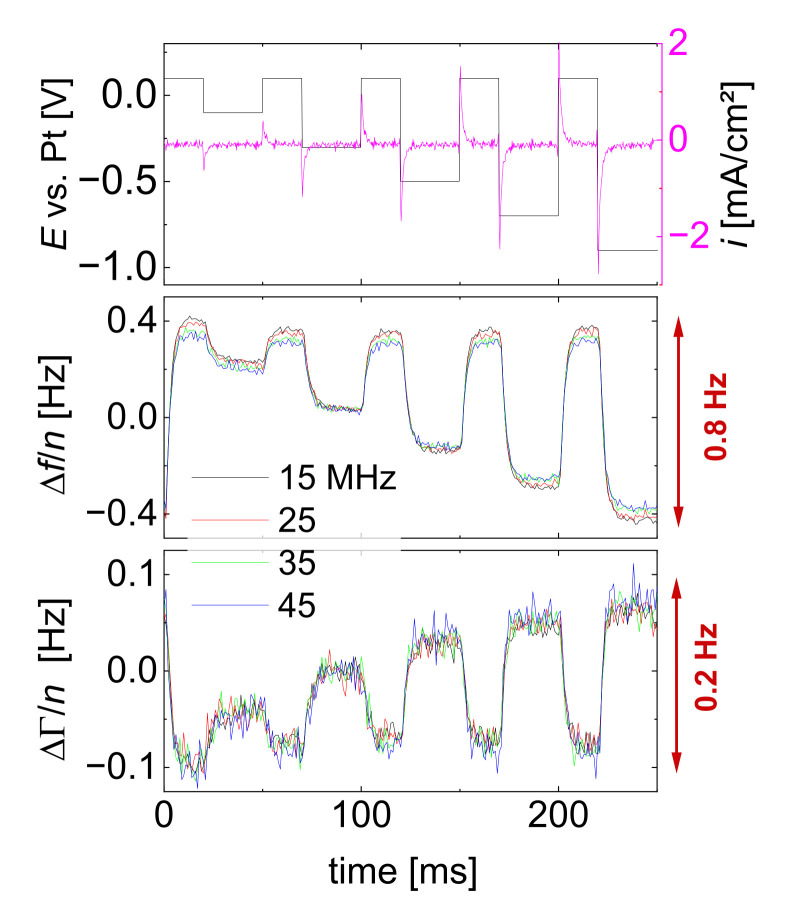
An example, where an EQCM responds to changes in a liquid’s near-surface viscosity. A solution of electrochemically inert ions was subjected to voltage steps, as shown at the top. The current recharges the diffuse double layer. The shifts in frequency are much larger than the shifts in bandwidth, and Δ*f*/*n* is similar on the different overtones. Typically, such a Sauerbrey-type behavior would be interpreted as being caused by adsorption, but increased viscosity in the diffuse double layer is equally possible. The kinetics suggests that altered viscosity makes a larger contribution to the overall frequency shift than adsorption. For details, see ref. [28]. The nonzero shift in ΔΓ shows that there is a small elastic component in the double layer’s response. Adapted from ref. [28].

Figure 6 shows an experimental example [28]. Inert electrolytes were employed, which do not undergo redox reactions at the electrode. Surprisingly, the data traces were strictly always of the Sauerbrey type (−Δ*f* >> ΔΓ and −Δ*f*/*n* ≈ const.). This finding can be explained by writing Equation (10) in terms of $\tilde{\eta }$ rather than $\tilde{J}$:

(16) $$\begin{array}{l} \frac{\Delta f+i\Delta \Gamma }{n}\approx -\frac{2f_{0}^{2}}{Z_{q}}\rho_{f}d_{f}\left[1-\frac{i\omega \rho_{bulk}}{i\omega \rho_{f}{\tilde{\eta }}_{f}}\right]\\ \approx -\frac{2f_{0}^{2}}{Z_{q}}\rho d_{f}\left[\frac{{\tilde{\eta }}_{f}-\eta_{bulk}}{{\tilde{\eta }}_{f}}\right] \end{array}$$

In the second step, it was assumed that the density of the near-surface layer is similar to the density of the bulk. This approximation was made for the sake of simplicity and is actually questionable in electrochemistry. (Variable density can be retained in the equations, straightforwardly.) We write $\tilde{J}$_f_ as η_bulk_ + Δ$\tilde{J}$ and assume that |$\Delta \tilde{J}$_f_| << η_bulk_:

(17) $$\frac{\Delta f+i\Delta \Gamma }{n}\approx -\frac{2f_{0}^{2}}{Z_{q}}\rho d_{f}\left[\frac{\Delta \tilde{\eta }}{\eta_{bulk}+\Delta \tilde{\eta }}\right]\approx -\frac{2f_{0}^{2}}{Z_{q}}\rho d_{f}\left[\frac{\Delta \tilde{\eta }}{\eta_{bulk}}\right]$$

The consequences are twofold:

- Sauerbrey-type behavior occurs if Δ$\tilde{\eta }$ is mostly real (that is, if the double layer has increased Newtonian viscosity with negligible viscoelasticity). Double layer effects cannot be distinguished from adsorption. In ref. [28] it was argued that the distinction is possible based on the kinetics of the response to a voltage step. The distinction is not possible based on single sets of Δ*f*/*n* and ΔΓ/*n*.
- The term in square brackets will often be smaller than unity. The thickness of the layer with increased viscosity can no longer be inferred from −Δ*f*/*n*, meaning that the gravimetric information is lost. If η’ depends on *z* (which can be expected), the product *d*_f_(η_f_′ − η_bulk_) turns into an integral ∫(η_f_′ − η_bulk_) d*z*.

The above discussion focused on the diffuse double layer from electrochemistry, but a similar problem exists for the dilute adsorbate (case D in Figure 1). These adsorbates can be soft to the extent that they form a layer with increased Newtonian viscosity. Differing from the diffuse double layer, they can be many nanometers thick.

We slightly digress in the following and discuss how the acoustic ratio is related to the viscoelastic parameters of the film. In a discussion of the diffuse double layer, the interpretation of the acoustic ratio with Equation (15) is inconvenient, because Equation (15) contains *J*_f_′ and *J*_f_″. For liquids, the absolute value of the viscosity, |$\tilde{J}$|, and the loss tangent, tan δ = η′_f_/η″_f_, are the more suitable parameters. (Arguably, an even more appropriate choice would be |${\tilde{J}}_{f}$|and the inverse loss tangent, because the inverse loss tangent is zero for a Newtonian liquid.)

Expressed in terms of |$\tilde{J}$| and tan δ, Equation (15) turns into the following:

(18) $$\frac{\Delta \Gamma }{-\Delta f}=\frac{\frac{\eta_{bulk}}{\left|{\tilde{\eta }}_{f}\right|}}{\sqrt{1+{\left(\tan\delta \right)}^{2}}(1-\frac{(\frac{\eta_{bulk}}{\left|{\tilde{\eta }}_{f}\right|})\tan\delta }{\sqrt{1+{\left(\tan\delta \right)}^{2}}})}$$

In the limit of tan δ → ∞ (an almost Newtonian liquid), the acoustic ratio is as follows:

(19) $$\frac{\Delta \Gamma }{-\Delta f}\approx \frac{1}{\tan\delta }\frac{1}{\frac{\left|{\tilde{\eta }}_{f}\right|}{\eta_{bulk}}+1}\approx \frac{1}{2\tan\delta }$$

If |$\tilde{J}$_f_| is similar to η_bulk_, the acoustic ratio is about half of the inverse loss tangent.

### 3.5. The Soft Adsorbate (Case D in Figure 1)

**Figure 7:**
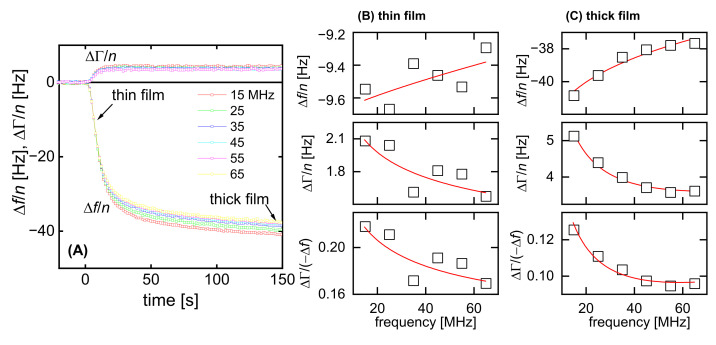
Adsorption of a polymer brush as an example, where the material parameters cannot be determined without making assumptions (early in the adsorption process, “thin film”), but where such an analysis becomes possible once the thickness exceeds 10 nm (“thick film”); the fractional noise decreases correspondingly (**A**). The raw data in (**A**) have been pre-averaged. Every data point is an average of four adjacent points of the raw data. The bottom panels in (**B**,**C**) show the acoustic ratios. The fits are not based on the acoustic ratios, but rather on Δ*f*/*n* and ΔΓ/*n* themselves. The fit was produced with the PyQTM program by using Equation (3).

The soft adsorbate, at the same time, is the general case, in which no assumptions on the viscoelasticity of the layer can be made. Figure 7 shows a dataset which, firstly, illustrates the problems with interpreting QCM data taken from such soft thin layers and, secondly, illustrates that these problems are less severe once the layer reaches a thickness of 10 nm or more. The data were taken while adsorbing a diblock copolymer with a hydrophobic anchor and a hydrophilic buoy to the resonator surface, where the latter had been coated with polystyrene (PS). Details are unessential. For the context of this work, see ref. [29].

**Figure 8:**
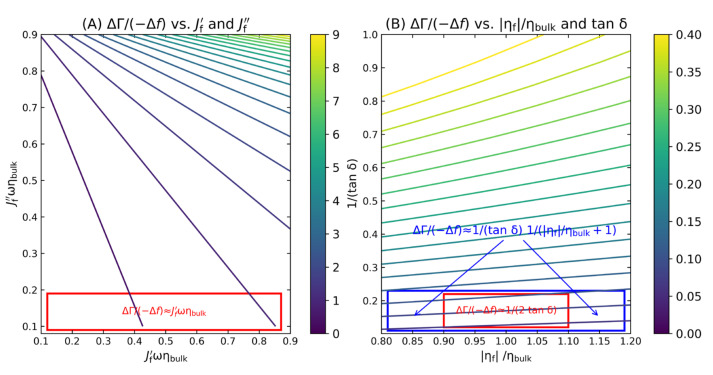
Contour plots of the acoustic ratio versus the viscoelastic parameters. Combinations of values on the contour lines all lead to the same acoustic ratio. The bars indicate the regimes, in which the assumptions underlying Equation (12) (**A**) and Equation (19) (**B**) apply.

**Figure 9:**
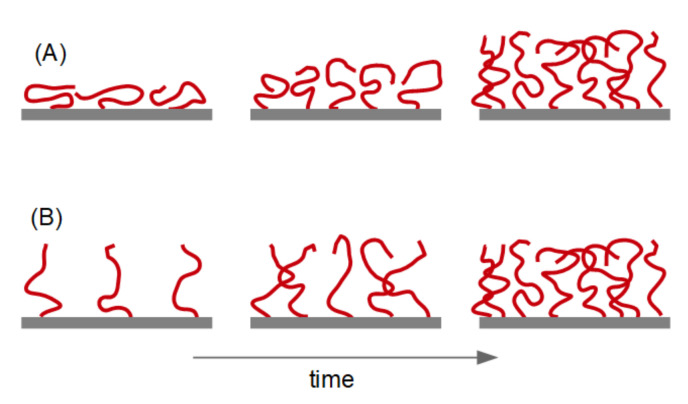
The QCM cannot determine whether an adsorbing polymer layer is initially thin and compact (**A**) or extended and dilute (**B**). The thickness in the later stages can be determined. The sketch is not meant to say that the configurations on the left-hand side (top and bottom) would lead to the exact same values of Δ*f*/*n* and ΔΓ/*n*; if that was the case QCM-D could determine the coverage, which it cannot.

Naively interpreted with the Sauerbrey equation, the data point at *t* ≈ 5 s (Figure 7B) would correspond to a layer thickness of 2 nm. To arrive at this result, divide −Δ*f*/*n* by 5.7 Hz/nm (assuming that ρ_f_ = 1 g/cm^3^); however, it is far from certain (and even unlikely) that 2 nm is actually the geometric thickness at this time. There are two problems:

- Applying the Sauerbrey equation implicitly assumes a rigid layer. If no assumptions on the viscoelastic properties of the layer can be made, a wide range of viscoelastic constants—if combined suitably—can match an experimentally determined acoustic ratio. Figure 8 shows the predictions of Equations (15) and (18) as contour plots (A and B). Any set of parameters on a contour line will lead to the same value of ΔΓ/(−Δ*f*). Only if additional assumptions are made (red boxes in Figure 8A) can the acoustic ratio be interpreted, e.g., in terms of Equation (12) (in the limit of small *J″*) or Equation (19) (in the limit of tan δ ≈ ∞ and η_f_′ ≈ η_bulk_′ blue and red boxes in Figure 8B).
- This uncertainty in the determination of viscoelastic parameters often spills over to the thickness. The point here is that the QCM cannot distinguish between a compact stiff layer and a dilute soft layer. The problem is illustrated in Figure 9.

The situation has much improved at *t* = 150 s (Figure 7C). Because the overall negative frequency shift has now increased to 40 Hz, the fractional noise is small enough to quantify the curvature reliably. This thickness of the sample is outside the range defined as “thin” in Section 3.3 and Section 3.4. The data were therefore not interpreted with the recipe from Section 3.3, but rather fitted with Equation (3). The software package PyQTM was used (Section 4). The parameters corresponding to the fit, shown in Figure 7C, are *d*_f_ = 9.8 nm, *J*_f_′ = 0.29 MPa^−1^, β′ = −1.61, *J*_f_″ = 1.68 MPa^−1^, and β″ = −0.91.

**Figure 10:**
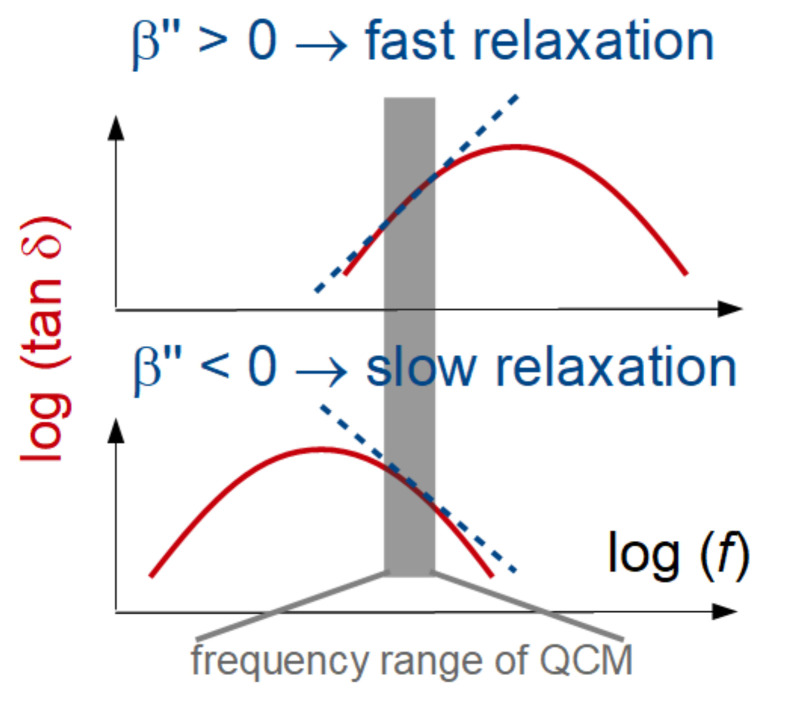
Viscoelastic spectra (red lines) and their approximation with power laws in a limited frequency range (dashed lines). The sign of the power law exponent pertaining to tan δ indicates whether the main relaxation is faster or slower than the frequency of the QCM. Β″ in the legend is the power law exponent of tan δ.

A side remark: If all five model parameters can be obtained with moderate errors, it is instructive to use tan δ as one of the fit parameters. Underlying viscoelasticity are relaxations, where the peak in tan δ corresponds to a typical rate. If β″ (the power law exponent of tan δ, cf. Equation (1)) is positive, the peak in tan δ is at frequencies above the frequency range of the QCM (top in Figure 10). The relaxations then occur at times above 1/(60 × 10^7^ s^−1^) ≈ 20 ns. If fitted with |$\tilde{G}$|and tan δ, the fit parameters leading to Figure 7C are *d*_f_ = 8.1 nm, |$\tilde{G}$_f_| = 1.0 MPa^−1^, β′ = −1.8, tan δ = 1.8 MPa^−1^, and β″ = −0.7. β″ is positive, indicative of fast relaxations.

**Figure 11:**
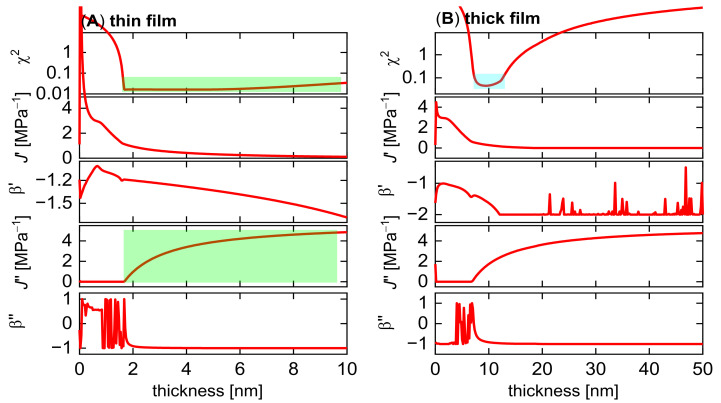
An analysis of the χ^2^ landscape for the datasets shown in Figure 7B,C. The software prescribes values for the thickness in a certain range (0 to 10 nm in (**A**) and fits the remaining parameters, which are *J*_f_′, β′, *J*_f_″, and β″. Given that the thickness is no longer a free parameter, χ^2^ is larger than what is obtained with *d*_f_ as a free parameter. As the top panel in (**A**) shows, χ^2^ is very large when *d*_f_ is smaller than 2 nm, but is only marginally larger than the minimum value when *d*_f_ is larger than 2 nm. The fit compensates for a large thickness with a large *J*′ and finds a good match with the experimental data (green bars on the left-hand side). The algorithm cannot distinguish between films that are either thin and stiff or thick and soft. The valley in the χ^2^ landscape is not at all sharp, and a statement on the thickness is difficult. These problems are much alleviated in (**B**) because the fractional errors have decreased in Figure 7C compared to Figure 7B. The valley in the χ^2^ landscape is now sharp (top in (**B**), light blue bar). On a qualitative level, the data now allow for a robust statement on the curvatures (Figure 7C). The interpretation can rest on a total of six robust experimental parameters (offsets, slopes, and curvatures in plots of Δ*f*/*n* and ΔΓ/*n* versus n.) Five model parameters can be inferred from six experimental parameters. The fit problem is now overdetermined (as it should be).

Figure 11 shows an analysis of the uncertainty for the derived thickness. The algorithm varies the film thickness, minimizes χ^2^ by using the remaining fit parameters, and plots the values of χ^2^ and the fitted other parameters versus thickness. As Figure 11A shows, χ^2^ sharply increases when the film thickness is lowered from its optimum value to below 1.8 nm. A film thickness of less than 1.8 nm is incompatible with the experiment; however, the increase in χ^2^ is rather moderate when *d*_f_ is moved upward into a range between 2 nm and 10 nm. The algorithm finds a good match with the experiment by compensating for a large thickness with a large *J*’ (grey bars in Figure 11A). No such problem occurs in Figure 11B. The χ^2^ landscape now has a distinct minimum at *d*_f_ ≈ 10 nm. This film thickness is now a robust outcome of the fitting process. Again, the result is robust because the noise is low enough to let the curvatures in Figure 2C be quantified with confidence.

In principle, one might hope to derive the coverage rather than the geometric thickness if the latter has large error bars. That would be possible if the second term in Equation (9) was inversely proportional to the local density (often the polymer volume fraction, ϕ). There usually is a monotonous relation between the two (dilute adsorbates are soft adsorbates), but the quantitative details are uncertain. A similar problem exists for the optical methods of thickness determination. In SPR spectroscopy, the signal (in “refractive index units”, RIUs) is related to the adsorbed amount, but the conversion from RIU into adsorbed amount is difficult [30]. If it was easy, RIUs as units would have disappeared. Both in optics and in shear wave acoustics, there is a poorly understood contrast function. The matter is discussed in more detail in Section 9.2. of Ref. [5].

In Figure 11A, the uncertainty in the knowledge on viscoelastic parameters has caused a corresponding uncertainty in terms of thickness. We have seen examples to the contrary. In some cases, one of the viscoelastic parameters had a shallow valley in the χ^2^ landscape, while the thickness was still well-defined. These questions need attention to detail in every single case.

## 4. The Software Package PyQTM

PyQTM is an open source software package for analyzing QCM-D data acquired with various platforms (Biolin Scientific QSoft, AWSensors AWSuite, or Clausthal QTZ). At its core is Equation (3), expanded to account for the possibility of two layers rather than one. It is written in Python and is available for download at https://www.pc.tu-clausthal.de/en/research/qcm-modelling/, source code included. Its graphical user interface allows users to choose between different representations of viscoelasticity, such as {*G*′, *G*″}, {*J*′, *J*″}, {|$\tilde{G}$|, tan δ}, and others. Furthermore, PyQTM includes a module which calculates χ^2^ landscapes for evaluating the robustness of the fit parameters against experimental noise and, also, checks for the influence of a fuzzy interface between the film and the bulk. Figure 7B,C, as well as Figure 11, were created using PyQTM.

## 5. Conclusions

A layer’s viscoelastic parameters can be extracted from the QCM data taken from thin films if the ambient medium is air or if the sample is a stiff film in a liquid, where “stiff” amounts to an assumption. For the case of a diffuse double layer in electrochemistry (a layer with altered viscosity), the acoustic ratio, ∆Γ/(−∆f), is proportional to the inverse loss tangent in this layer. In this case, a statement on layer thickness cannot be made. For the general case, the problem is underdetermined because the model has five parameters, while the QCM data can be aggregated into only four parameters. Once the film thickness exceeds about 10 nm, even soft layers can be analyzed, because the analysis can then also exploit the curvature in plots of −∆f/n and ∆f/n versus *n*. Five model parameters can then be reliably derived from six robust parameters contained in the sets of ∆*f*/*n* and ∆Γ/*n*.

## Data Availability

Data available on request.
